# A250 NUTRISUP-PPN; PILOT RANDOMIZED CONTROL TRIAL OF ORAL AND PARENTERAL NUTRITION IN MALNOURISHED HOSPITALIZED PATIENTS

**DOI:** 10.1093/jcag/gwab049.249

**Published:** 2022-02-21

**Authors:** A Rahman, M Mrkobrada, A Patel, A Chakroborty, H Stephanie, D Armstrong

**Affiliations:** 1 Gastroenterology, Western University, London, ON, Canada; 2 McMaster University, Hamilton, ON, Canada

## Abstract

**Background:**

Hospitalized malnourished patients experience poor outcomes. Despite this, health care practitioners are poor in identifying, monitoring and treating hospital malnutrition.

**Aims:**

Our study determined the feasibility of a novel nutrition care pathway which both rapidly identifies and treats malnourished medical inpatients accounting for the obstacles for nutritional optimization by utilizing both peripheral parental nutrition (PPN) followed by oral nutritional supplementation (ONS) on a composite outcome of 30 day readmission, mortality and continued admission, as well other important clinical and nutritional outcomes. The study was registered under ClinicalTrials.gov Identifier no. NCT02632630

**Methods:**

NutriSUP-PPN was a 2x2 factorial pilot randomized trial. In two large Canadian hospitals, we recruited 100 adult patients > 18 years, < 48 hours from admission to a general medicine ward who were moderately or severely malnourished. Patients received: 1. PPN for 5 days and then enhanced ONS until 30 days post randomization; 2. PPN for 5 days and then standard ONS until 30 days; 3. Standard care for intravenous (IV) fluid administration for 5 days and then enhanced ONS until 30 days; 4. Standard care for IV fluid administration for 5 days and standard ONS until 30 days.

**Results:**

There was no significant differences between a composite outcome of 30 day readmission, continued admission or mortality between any interventional group and control. We did however note a trend in the PPN + ONS arm where only 4/22 patients versus 10/24 patients (p=0.16) in the control (no PPN, no enhanced ONS) experienced an adverse outcome which was largely driven by a reduction of readmission in the ONS + PPN arm We demonstrated feasibility in recruitment, adherence to protocol, and safety. The incidence of sepsis was greater in the PPN arm compared to control (15.5% versus 4.2%) but was not statistically significant. Improvement in nutritional status for interventional arms were not significant compared to control. However, there was a trend of improvement in preventing decline of nutritional status in both the enhanced ONS arm and PPN + enhanced ONS arm.

**Conclusions:**

There are signals in our data, which suggest that the combination of PPN with ONS may improve both clinical and nutritional outcomes compared to PPN or ONS alone. We posit that a large, multi-center, definitive randomized control trial is now justified to determine if PPN for up to 5 days along with 30 days of ONS, versus standard of care, will improve a composite outcome of death, continued admission, and readmission at 30 days.

**Funding Agencies:**

CAGAMOSO

